# Molecular Mechanism for the Thermo-Sensitive Phenotype of CHO-MT58 Cell Line Harbouring a Mutant CTP:Phosphocholine Cytidylyltransferase

**DOI:** 10.1371/journal.pone.0129632

**Published:** 2015-06-17

**Authors:** Lívia Marton, Gergely N. Nagy, Olivér Ozohanics, Anikó Lábas, Balázs Krámos, Julianna Oláh, Károly Vékey, Beáta G. Vértessy

**Affiliations:** 1 Institute of Enzymology, Research Centre for National Sciences, HAS, Budapest Hungary; 2 Doctoral School of Multidisciplinary Medical Science, University of Szeged, Szeged, Hungary; 3 Department of Applied Biotechnology and Food Science, Budapest University of Technology and Economics, Budapest, Hungary; 4 Institute of Organic Chemistry, Research Centre for National Sciences, HAS, Budapest, Hungary; 5 Department of Inorganic and Analytical Chemistry, Budapest University of Technology and Economics, Budapest, Hungary; University of Rome Tor Vergata, ITALY

## Abstract

Control and elimination of malaria still represents a major public health challenge. Emerging parasite resistance to current therapies urges development of antimalarials with novel mechanism of action. Phospholipid biosynthesis of the Plasmodium parasite has been validated as promising candidate antimalarial target. The most prevalent *de novo* pathway for synthesis of phosphatidylcholine is the Kennedy pathway. Its regulatory and often also rate limiting step is catalyzed by CTP:phosphocholine cytidylyltransferase (CCT). The CHO-MT58 cell line expresses a mutant variant of CCT, and displays a thermo-sensitive phenotype. At non-permissive temperature (40°C), the endogenous CCT activity decreases dramatically, blocking membrane synthesis and ultimately leading to apoptosis. In the present study we investigated the impact of the analogous mutation in a catalytic domain construct of *Plasmodium falciparum* CCT in order to explore the underlying molecular mechanism that explains this phenotype. We used temperature dependent enzyme activity measurements and modeling to investigate the functionality of the mutant enzyme. Furthermore, MS measurements were performed to determine the oligomerization state of the protein, and MD simulations to assess the inter-subunit interactions in the dimer. Our results demonstrate that the R681H mutation does not directly influence enzyme catalytic activity. Instead, it provokes increased heat-sensitivity by destabilizing the CCT dimer. This can possibly explain the significance of the *Pf*CCT pseudoheterodimer organization in ensuring proper enzymatic function. This also provide an explanation for the observed thermo-sensitive phenotype of CHO-MT58 cell line.

## Introduction

With 1.2 billion people being at high risk of infection, malaria still presents a major health challenge [1]. Development of antimalarials with novel mechanism of action is essential to keep pace with the emerging antimalarial drug resistance [2]. Targeting the lipid biosynthesis of the causative agent Plasmodium parasites is among the promising candidate antimalarial strategies [3]. It relies on almost exclusive use of phospholipids (PL) acquired by *de novo* biosynthesis as membrane constituents during the intraerythrocytic life stage of the parasite [4]. The choline analogue lead compound Albitiazolium was shown to block the carrier mediated choline entry into the parasite, besides, inhibition of further metabolic steps of the Kennedy phospholipid *de novo* phosphatidylcholine (PC) biosynthesis pathway were also confirmed [5].

Among the enzymes that assist PC biosynthesis in the parasite, CTP:phosphocholine cytidylyltransferase (CCT) is of particular interest. It catalyzes one of the rate limiting steps of the metabolic pathway by converting CTP and choline-phosphate (ChoP) to CDP-choline (CDPCho) and pyrophosphate (PP_i_). Besides, this enzyme is regulated by a reversible membrane interaction mechanism that involves structural rearrangement of two putative amphipathic α-helices in the membrane binding domain [6,7]. The lipid composition dependent membrane interaction results in 5.5–fold enzyme activity stimulation [8]. Truncated constructs of CCT from Plasmodium and rat consisting only the catalytic domain were shown to be constitutively active [9–11]. Essential role of CCT was demonstrated by gene disruption or knock-out experiments in Plasmodium [12] as well as several eukaryotic organisms [13–15] or cell lines [16–19].

A chemically mutated Chinese Hamster Ovary (CHO) cell-line with an inducible CCT-deficient phenotype was described as a tool for the functional investigation of CCT [19]. At a permissive temperature of 33°C, CHO-MT58 cells grow at a rate of about 80% of the parental line while maintaining 80–90% PC levels of the parental CHO-K1 strain [19]. At a non-permissive temperature of 40°C, the PC content of the mutant cells decrease by 40% in the first 8 h and by a total of 80% in 24 h [20] as a result of dramatically decreased CCT enzyme activity and CDPCho metabolite levels [19], eventually leading to apoptosis [21]. Noteworthy, mutant cells possess 20-fold less CCT activity than CHO-K1 cells even at 33°C. Western blot analysis demonstrated that the CCT content of CHO-MT58 cells is less than 5% of the amount in parental cells, while the respective steady-state mRNA levels are similar in the two cell types [22]. These results may suggest that the mutant CCT enzyme possesses impaired thermal stability, however this suggestion has not yet been experimentally verified. The temperature-sensitive phenotype of CHO-MT58 cells is conveyed by a single point mutation of the CCTα isoform [19,23,24]. The guanine to adenine nucleotide change at the position 419 corresponds to the point mutation R140H in the catalytic domain of the endogenous CCT.

High conservation of CCT catalytic domains enables the investigation of the role of R140 residue in the rat CCT crystal structure [25]. Similarly to the majority of CCT enzymes, rat CCTα functions as a homodimer [26,27]. R140 is part of ^140^RYVD^143^ sequence motif that has key importance in dimer stabilization in case of rat CCT [25]. Crystal structures show that this segment, buried at the dimer interface, anchors the two monomers with the arginine forging multiple inter-chain polar interactions [25,28].

Although the point mutation in the *cct* gene was already described in 1994 and the cell line CHO-MT58 is well characterized and frequently used in studying apoptosis and lipid metabolism (for example [29–32]), still there are no direct *in vitro* enzyme studies to describe the molecular mechanism causing this thermo-sensitive phenotype.

In the present study we investigated the *in vitro* effects of the mutation corresponding to R140H in the catalytic domain construct of *Plasmodium falciparum* CCT. *In silico* and *in vitro* R/H mutagenesis in the *Pf*CCT catalytic domain enabled the investigation of the consequences of the mutation on enzyme structure, function and stability. Our results highlight the significance of *Pf*CCT catalytic domain dimer formation and reveal that the quaternary structure has critical role in ensuring enzyme function. This contributes to the molecular characterization of this important antimalarial drug target enzyme. Besides our *in vitro* study provides a probable explanation for the thermo-sensitive phenotype observed in CHO-MT58.

## Materials and Methods

### Materials

Restriction enzymes and DNA polymerases were obtained from New England Biolabs (Ipswich, MA, USA). Isopropyl β-D-1-thiogalactopyranoside (IPTG) was obtained from Fisher Scientific GmbH (Schwerte, Germany). Nickel-nitrilotriacetic acid (Ni-NTA) was from Qiagen (Düsseldorf, Germany), protease inhibitor cocktail tablets were purchased from Roche (Basel, Switzerland). CTP, CDPCho, Sypro Orange, inorganic pyrophosphatase, purine nucleoside phosphorylase, DNA purification kit and antibiotics were purchased from Sigma-Aldrich (St Louis, MO, USA). Phosphocholine chloride sodium salt hydrate (further termed as ChoP) was from TCI Europe N.V. (Antwerp, Belgium). MESG (7-methyl-6-thioguanosine) was obtained from Berry and Associates (Dexter, MI, USA). All other chemicals were of analytical grade of the highest purity available.

### Alignment of CCT sequences

Conservation of the RYVD motif was investigated using the PipeAlign webserver [33]. The PipeAlign is a protein family analysis method using a five step process beginning with the search for homologous sequences in protein and 3D structure databases and ending up in the definition of subfamilies (clusters). The server performs multiple alignment of 200 complete sequences originating from different clusters. Blast search for homologous sequences of rat CCTα, (Uniprot code: P19836) was performed using Ballast with filter for BlastP search, then Blast gapped alignment was done on 200 sequences from sampled Blast/Ballast results with fragments removal, then adjusted manually. Selected sequences were chosen from different clusters using the most appropriate clustering method suggested by the server. The extent of conservation of selected amino acids was visualized by Weblogo [34].

### Homology modelling and molecular dynamics simulations

**Fig 1:**
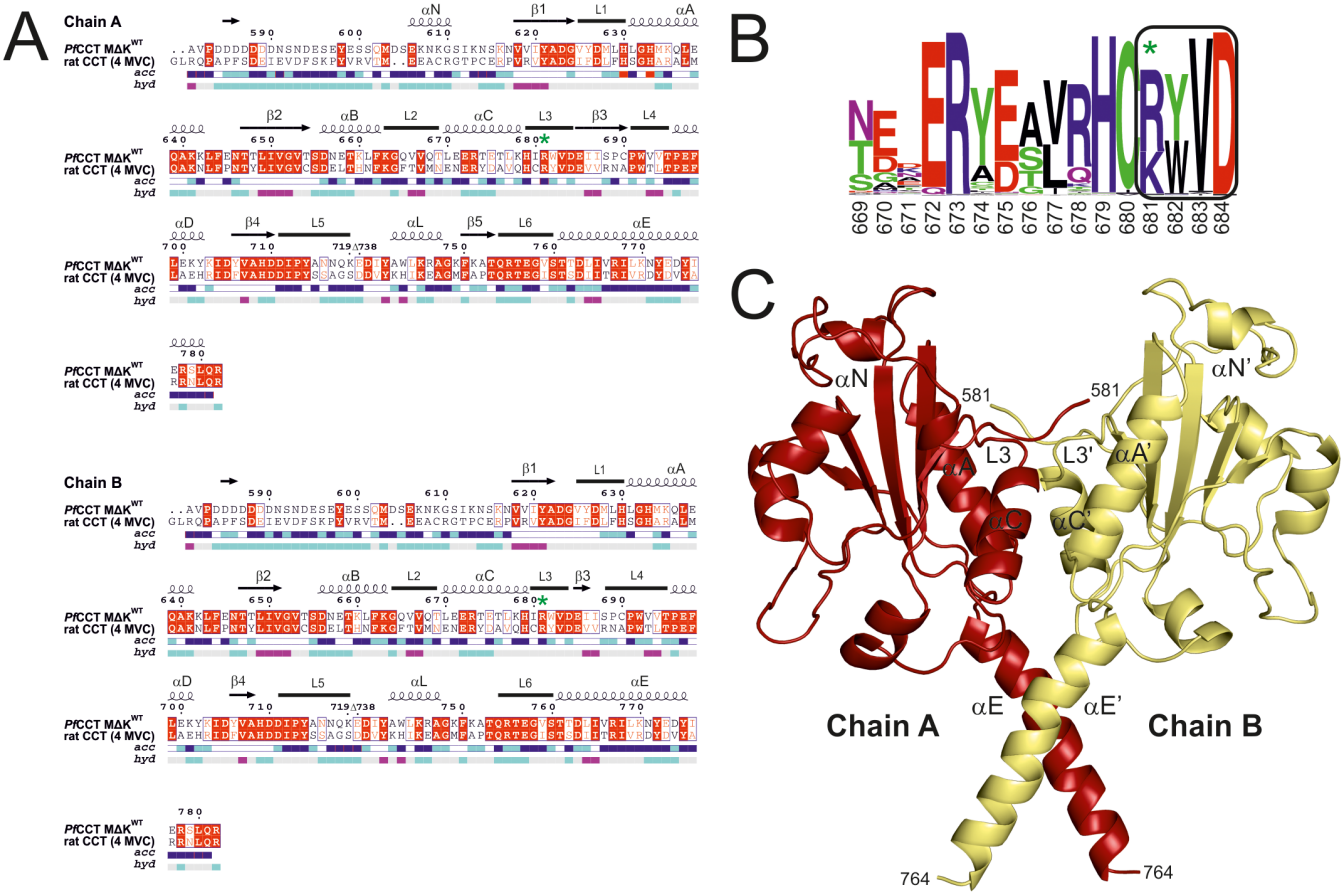
Protein structure prediction of *Pf*CCT MΔK^WT^ and *Pf*CCT MΔK^R681H^. A) Alignment of rat CCT (PDB ID: 4MVC) and *Pf*CCT MΔK^WT^ sequences used for modeling and MD stimulations. Numbering is according to *Pf*CCT MΔK^WT^. Secondary structure elements are represented by squiggles (α-helices), arrows (β-strands) and lines (turns). In the aligned sequences, red box with white character indicates strict identity and red character means similarity in groups. R681 corresponding to R140 in rat CCT is indicated by a green star. Hydropathy (pink—hydrophobic, grey—intermediate, cyan—hydrophilic) and accessibility (blue—accessible, cyan—intermediate, white—buried) are also presented below the sequences. The layout with secondary structure elements was generated with ESPript 3.0 [66], supplemented with visual inspection of structures. B) Conservation of the RYVD signature sequence in CCTs, shown by Weblogo. Numbering is according to the *Plasmodium falciparum* sequence, where the ^681^RWVD^684^ corresponds to the ^140^RYVD^143^ in the rat sequence. C) Dimer structure of MΔK^WT^ homology model. Chain A is coloured in red and chain B is coloured in yellow. Important secondary structure elements are indicated.

The catalytic domain of the rat CCT (PDB ID: 4MVC) [35] was used to construct the homodimer homology models of *Pf*CCT MΔK^WT^ containing the second catalytic domain of *Pf*CCT (528–795, Δ720–737) and its point mutant *Pf*CCT MΔK^R681H^. The aligned sequences were 44.56% and 44.02% identical in the case of wild type and R681H mutant CCT, respectively [36] (Fig 1A). MODELLER 9.14 [37] software was used to create 80 homology models in both cases using the same alignment. The models with the lowest value of the MODELLER objective function were selected and visually inspected using VMD program [38]. The selected models were evaluated using PROCHECK [39], WHAT_CHECK [40] and ERRAT [41] programs (for further details see S1 File). Model data are available in the Protein Model DataBase (PMDB) under the accession number PM0079950 (*Pf*CCT MΔK^WT^) and PM0079951 (*Pf*CCT MΔK^R681H^).

Molecular dynamics (MD) simulations were carried out for both enzyme variant models using the same computational protocol. The protonation state of the ionisable amino acid side chains was verified by H++ webserver version 3.1 [42] and PROPKA [43]. Based on the estimated pKa values residues D584, D585, E685 of chain A and D584, E685 of chain B were protonated in both enzyme variants. In addition, D589 and D590 were also protonated in chain B of *Pf*CCT MΔK^R681H^. Based on the pKa predictions H709 and H681 at the mutated position were protonated both on the δ and ε positions, having a +1 charge. The surrounding of neutral histidine residues was visually inspected to decide their most likely protonation state, and all histidine residues were protonated in the ε position in both enzyme variants with the exception of H679 which was as protonated on the δ position. CHARMM program [44] and CHARMM27 force field [45] was applied using the self-consistent GBSW implicit solvent model [46] to carry out the MD simulations. The calculations were carried out with the optimized PB radii [47], and the CMAP correction optimized for GBSW [48]. The nonpolar surface tension coefficient was 0.005 kcal/(mol Å^2^), the number of angular integration points was 50 and the grid spacing for lookup table was 1.5 Å. Structures were heated up from 10 K to 310 K over 60 ps. At this temperature MD equilibration was carried out over 100 ps, which was followed by the final 5 ns long productive MD simulation. Interaction energies between the two chains of enzyme variants were calculated over the whole trajectory for all frames (i) defined as Eq (1):

$$W{\left(R^{M}\right)}_{\text{int}}^{i}=W{\left(R^{M}\right)}_{\text{dim}er}^{i}-W{\left(R^{M}\right)}_{chainA}^{i}-W{\left(R^{M}\right)}_{chainB}^{i}$$ (1)

where W(R^M^) is the effective energy of the protein with coordinates R^M^ in solution (Eq (2))

$$W(R^{M})=H_{\text{int}ra}(R^{M})+\Delta G_{solv}(R^{M})$$ (2)

where H_intra_ is the intramacromolecular energy, consisting of bonded and non-bonded energy terms, and ΔG_solv_ is the solvation free energy [49].

The volume and the surface of the proteins were calculated by 3v website [50] using a high resolution grid and 1.4 Å probe radius. The number of hydrogen bonds were measured by CHARMM using the default 2.4 Å distance and 999.0 angle cut-offs.

### Mutagenesis, protein expression and purification

The R681H mutant construct of His-tagged *Pf*CCT MΔK (528–795, Δ720–737) was produced by site-directed mutagenesis [10] (PlasmoDB accession number: PF3D7_1316600) [51] using the QuikChange method (Agilent) (for more details about sequence of the protein used for *in vitro* studies see Fig. A in S2 File). Primers used for mutagenesis (R681H 5’-3’, gaaacacatcCATtgggttgac; R681H 3’-5’, gtcaacccaATGgatgtgtttc) were synthesized by Eurofins MWG GmbH. Constructs were verified by DNA sequencing at Eurofins MWG GmbH. *Pf*CCT MΔK^WT^ and *Pf*CCT MΔK^R681H^ were expressed and purified as described previously [10] with minor modifications. Briefly, the His-tagged fusion proteins were expressed using the BL21 (DE3) Rosetta *E*. *coli* expression system. Expression was induced with 0.6 mM IPTG for 20 h at 16°C. In case of *Pf*CCT MΔK^R681H^ Ni-NTA affinity chromatography was performed at 18°C to maintain protein stability. Protein eluted from Ni-NTA column was dialyzed into 20 mM HEPES, pH 7.5 buffer, containing 100 mM NaCl (buffer A). Samples for MS analysis were further purified by size-exclusion chromatography (gel filtration) using a GE Healthcare ÄKTA system with a Superose12 column.

Protein concentrations were determined spectrophotometrically from the absorbance at 280 nm using a Nanodrop 2000c spectrophotometer (Thermo Scientific). Extinction coefficient 31400 M^-1^cm^-1^ as calculated on the basis of amino acid composition by using ProtParam server was used [52].

### Steady-state activity

Steady-state activity measurements were performed as described previously [10] in buffer A using a continuous coupled pyrophosphatase enzyme assay, which employs MESG (7-methyl-6-thioguanosine) substrate for colorimetric phosphate detection [53]. For heat inactivation, protein samples were incubated for 15 min in buffer A at various temperatures (10-60°C); enzyme activity was immediately measured at 20°C.

### Kinetic titrations

For CTP substrate titrations, CTP concentration was varied between 12 μM and 1.2 mM while ChoP concentration was kept at 5 mM. For ChoP substrate titrations, ChoP concentration was varied between 0.1 and 20 mM while CTP concentration was kept at 1 mM. Kinetic data were fitted with Eqs (3) and (4) (Michaelis–Menten equation and competitive substrate inhibition equation, respectively) using OriginPro 8 (OriginLab Corp., Northampton, MA, USA):

$$v=\frac{v_{\text{max}}\left[S\right]}{K_{M}+\left[S\right]}$$ (3)

$$v=\frac{v_{\text{max}}}{\left(1+\frac{K_{M}}{\left[S\right]}+\frac{\left[S\right]}{K_{i}}\right)}$$ (4)

in these equations, v is the reaction rate, v_max_ is the maximum velocity of the reaction, [S] is the concentration of the substrate and K_i_ describes the binding of a substrate molecule to the enzyme resulting in a decrease of the maximal reaction rate by half.

### Mass spectrometry

In the mass spectrometric study of protein complexes, a commercial Waters QTOF Premier instrument (Waters, Milford, MA, USA) equipped with electrospray ionization source (Waters, Milford, MA, USA) was used in the positive ion mode. Mass spectra were obtained under native conditions; namely, the ions were generated from aqueous 5 mM NH_4_HCO_3_ buffer solution (pH 7.2) containing the gel filtered *Pf*CCT MΔK protein constructs at 0.4 μM monomer concentration. These conditions allow transfer of the native protein complex present in the solution into the gas phase. The capillary voltage was 3600 V, the sampling cone voltage was 125 V and the temperature of the source was kept at 80°C, collision cell pressure was 3.38·10^-3^ mbar and ion guide gas flow was 15.00 ml/min. Mass spectra were recorded using the software MassLynx 4.1 (Waters, Milford, MA, USA) in the mass range 1000-5000 m/z as no signals could be detected above 5000 m/z. To ensure reproducible results, 3 samples originating from different expressions were measured for both *Pf*CCT MΔK^WT^ and *Pf*CCT MΔK^R681H^.

## Results

### R681 is highly conserved and serves dimer stabilization roles

As the ^140^RYVD^143^ segment is of prime importance in dimer stabilization in case of rat CCT, we decided to analyze the overall conservation pattern of this motif in CCT enzymes. By performing a Blast search with PipeAlign webserver [33] we compared 200 CCT sequences from different evolutionary clusters. Our results confirmed the previously proposed high degree of conservation [28] for this sequence motif (cf. boxed residues on Fig 1B). While the first and second position of the motif is characterized with conserved basic (R/K) and aromatic (Y/W) residues, the last two positions are exclusively occupied by V and D. RYVD is the most frequently occurring motif, apparent in ca. 50% of investigated sequences. We also found conserved histidine and cysteine residues directly adjacent to this motif, which were also shown to participate in the interaction network stabilizing the dimer of rat CCT [25]. Remarkably, none of the investigated sequences contained a histidine at the arginine position (noted by a star on Fig 1B), despite its potentially basic character.

Additionally, we performed a dbSNP database search for human pcyt1a corresponding CCTα to see whether this mutation is present as an amino acid variation. From the 183 single nucleotide variations denoted up to November 2014, 48 caused missense mutations and 2 nonsense mutations, but none of them concerned either the RYVD or HxGH motif, another signature sequence of cytidylyltransferases, that plays key role in catalysis [54]. Nevertheless, Payne et al. described the mutation of V142 in human CCT causing congenital lipodystrophy and fatty liver disease [55]. This residue is the main interaction partner of R140, therefore its exchange to methionine may adversely affect dimer interaction. These observations underline the importance of the integrity of RYVD motif.

**Fig 2:**
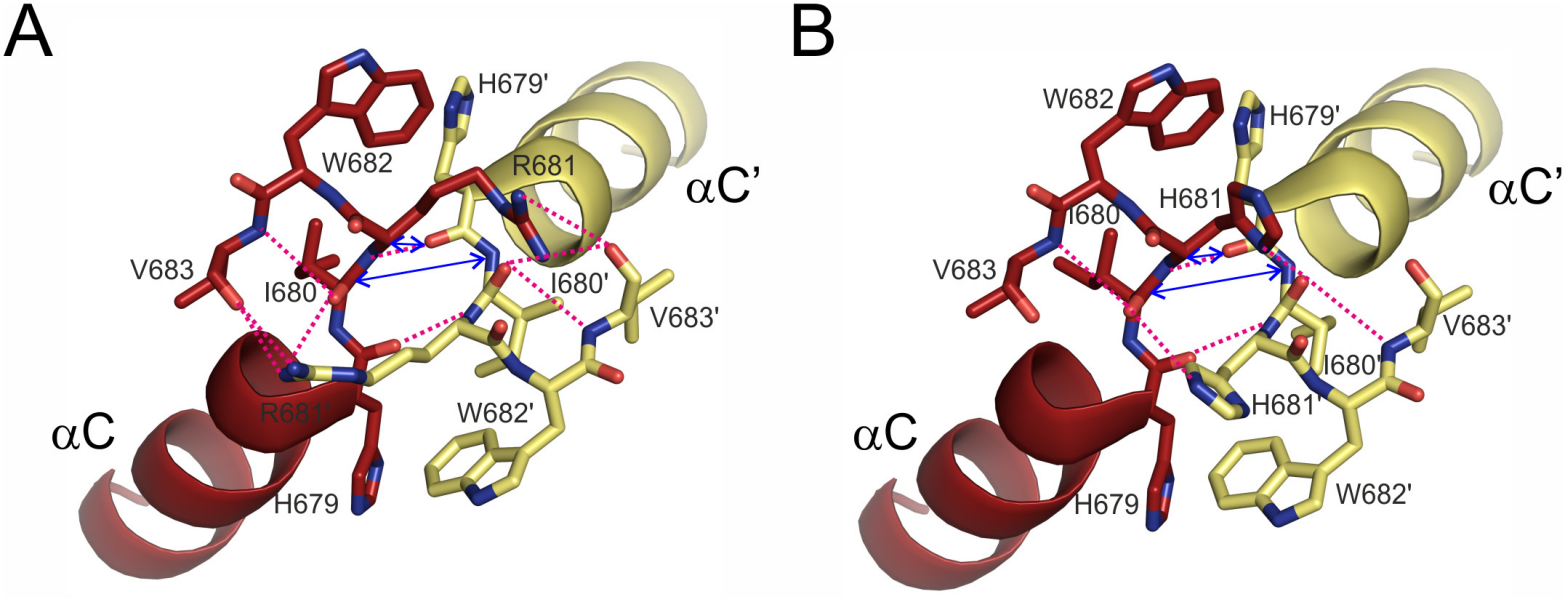
Polar interactions at the dimer interface of *Pf*CCT MΔK^WT^ and *Pf*CCT MΔK^R681H^ involving ^681^RWVD^684^. A) Direct interactions harbouring ^681^RWVD^684^ signature sequence motif in *Pf*CCT MΔK^WT^. The residues involved in the inter-chain interaction are shown in stick representation, and interactions are indicated by pink dashed lines. Characteristic dimer interface distances d(R681, CA - H679’, O) and d(I680, CA - I680’, N) are denoted by blue double-headed arrows. Residues in chain B are marked with apostrophes. B) Direct interactions harbouring the ^681^HWVD^684^ mutated signature sequence motif *Pf*CCT MΔK^R681H^. The residues involved in the inter-chain interaction are shown in stick representation, and interactions are indicated by pink dashed lines. Characteristic dimer interface distances d(H681, CA - H679’, O) and d(I680, CA - I680’, N) are denoted by blue double-headed arrows. Residues in chain B are marked with apostrophes.

Our *Plasmodium falciparum* CCT construct (*Pf*CCT MΔK) encompasses the second catalytic domain (Cat2) of the full length enzyme including ^681^RWVD^684^ as the analogue for the cognate motif (of ^140^RYVD^143^ in the rat sequence). CCT evolved in Plasmodia by a lineage-specific gene duplication event, resulting in duplicated catalytic and membrane binding domains [10]. Construct of the second catalytic domain of *Pf*CCT, termed as MΔK, was shown to exist in a dimer oligomerization state *in vitro* that is highly similar to the assembly of Cat1Cat2 catalytic domains from the full length enzyme [10]. To visualize inter-subunit interactions of MΔK dimers, a homology model was built using the rat CCT catalytic domain structure as a template [35] (Fig 1A and 1C). Due to the considerable sequence identity between target and template, the *Pf*CCT model displayed similar fold as the rat CCT. As in the case of rat CCT [25], R681 provides two direct inter-subunit polar interactions to the main chain atom of V683’ and one polar main chain interaction through I680 to V683’ (Table 1). There is also a polar main chain interaction present between the main chain atoms of I680 and R681’ bringing the two chains to a distance of 3.7 Å at this specific point (d(R/H681, CA - H679’, O)) (Fig 2A and 2B). Polar interactions of H679 and W682 (corresponding the H138 and Y141 in rat) are missing (Fig 2A). Overall, eight polar interactions can be identified that contribute to dimer stability in the Plasmodium CCT structural model. *In silico* modelling of the R681H mutation showed that the two direct interactions between V683 and R681 are lost (Table 1 and Fig 2B), which indicates a possibility for decreased inter-domain stability [56]. N-terminal segment contributes also to dimer stabilization by forging contacts with helix αA and loop L3, which is also effected by R681H mutation (Table 1).

**Table 1:** Polar inter-chain interactions between L3, αA and N-terminal regions in homology models of both enzyme variants.

*Pf*CCT MΔK^WT^			*Pf*CCT MΔK^R681H^		
**L3**	**L3’**	**d (Å)**	**L3**	**L3’**	**d (Å)**
H679-O	R681-N	2.98	H679-O	H681-N	3.00
R681-NH2	I680-O	2.96	H681-ND1	I680-O	3.50
R681-NH1	I680-O	3.54			
R681- NH2	R681-O	2.79	H681- ND1	H681-O	3.40
R681- NH2	V683-O	2.65			
R681-N	H679-O	2.96	H681-N	H679-O	2.81
**L3**	**αA’**	**d [Å]**	**L3**	**αA’**	**d [Å]**
H679- NE2	K635-NZ	3.31			
**L3**	**Nterm’**	**d [Å]**	**L3**	**Nterm’**	**d [Å]**
R681- NH2	D584-OD1	2.69			
R681- NH1	D584-OD1	3.28			
R681- NH2	D584-OD2	3.53			
W682-NE1	A581-O	2.58	W682-NE1	A581-O	2.58
**Nterm**	**L3’**	**d [Å]**	**Nterm**	**L3’**	**d [Å]**
A581-O	W682-NE1	2.77	A581-O	W682-NE1	2.69
V582-O	R681- NH2	2.98			

N-terminal region consists of residues 581–620 (cf. Fig 1A).

### Unaltered enzyme function with impaired heat stability due to R681H mutation

**Fig 3:**
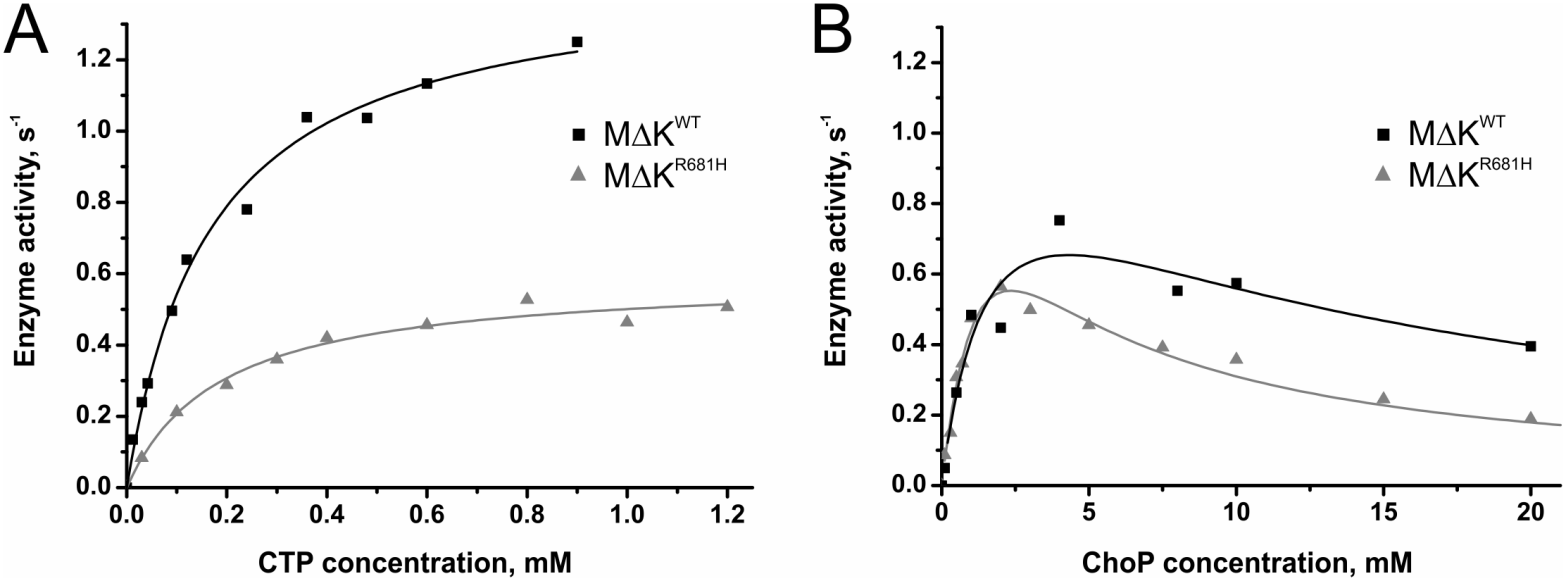
Steady-state kinetic analysis of *Pf*CCT MΔK^WT^ and *Pf*CCT MΔK^R681H^. A) CTP titration of the activity of the CCTs at a fixed ChoP concentration of 5 mM. The plot shows one representative experiment. Titration data are fitted with the Michaelis–Menten kinetic model assuming no cooperativity. B) ChoP titration of the activity of the CCTs at a fixed CTP concentration of 1 mM. The plot shows one representative experiment. Titration data are fitted with a kinetic model assuming substrate inhibition without cooperativity. Note the substrate inhibition effect of ChoP as an initial rate decrease is observed at higher substrate concentrations.

For *in vitro* studies we generated the R681H variant of the *Pf*CCT MΔK construct. Already upon expression of this variant performed at 16°C, we observed lower yield of expression as compared to the *Pf*CCT MΔK^WT^ (cf. Fig. A in S2 File). First, we investigated the functionality of *Pf*CCT MΔK^R681H^ at 20°C using a continuous spectrophotometric enzyme activity assay, and compared its kinetic properties to that of *Pf*CCT MΔK^WT^ [10]. Data shown in Fig 3 and Table 2 indicate that in the case of the mutant CCT enzyme the substitution to histidine attenuates k_cat_ by 60% when analyzed by [CTP] variation, but has little effect when analyzed by [ChoP] variation assayed at the permissive temperature of 20°C. In addition, substrate inhibition observed at millimolar ChoP concentration range is also apparent with the mutant enzyme. Therefore the mutation does not alter the enzymatic function heavily under the experimental conditions. These results are in good agreement with findings on the CHO-MT58 cell line, which was shown to possess a wild-type phenotype at permissive temperatures (33°C) despite reduced overall CCT levels [24].

**Table 2:** Kinetic parameters of *Pf*CCT MΔK^WT^ and *Pf*CCT MΔK^R681H^ catalysis.

	CTP titration		ChoP titration		
	k_cat_ (s^-1^)	K_M, CTP_ (mM)	k_cat_ (s^-1^)	K_M, ChoP_ (mM)	K_i, ChoP_ (mM)
***Pf*CCT MΔK** ^**WT**^ *	1.45 ± 0.05	0.17 ± 0.02	1.2 ± 0.4	1.8 ± 1.1	10.5 ± 7.5
***Pf*CCT MΔK** ^**R681H**^	0.59 ± 0.02	0.19 ± 0.03	1.2 ± 0.2	1.6 ± 0.4	3.8 ± 1.0

*data obtained from [10]

**Fig 4:**
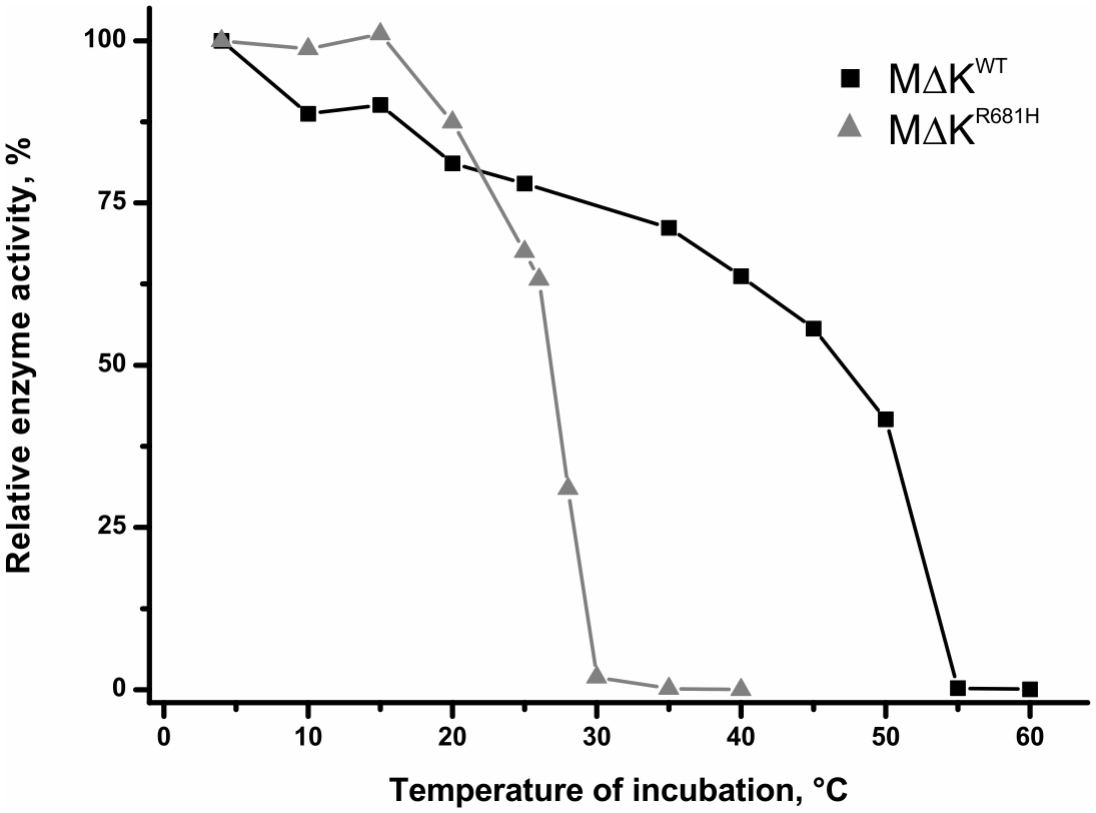
Kinetics of thermal inactivation of *Pf*CCT MΔK^WT^ and *Pf*CCT MΔK^R681H^. Protein samples were incubated for 15 min in buffer A at different temperatures prior to the measurement performed at 20°C. Inactivation is shown as the fraction of remaining CCT activity. One representative is shown for each temperature and each protein.

To elucidate the mechanism of temperature-induced inactivation of CCT, we characterized the thermal stability of *Pf*CCT MΔK^R681H^. We adopted the experiment described by Belužić et al. [57] to assess temperature dependence of protein stability and functionality. Protein samples were incubated at different temperatures for 15 minutes then their enzyme activity was measured immediately at 20°C. The mutant enzyme lost half of its activity at circa 25°C and was completely inactivated at 30°C, while similar relative thermal inactivation states of the wild type enzyme occurred at 55°C and 60°C, respectively (Fig 4). Importantly, the wild-type and mutant enzymes display a marked difference in kinetic stability at the physiological temperature range, which is in agreement with the temperature sensitivity of the CHO-MT58 strain.

### Perturbed oligomerization state and dynamic properties of *Pf*CCT MΔK^R681H^

Having demonstrated the drastically impaired thermal stability of the *Pf*CCT MΔK^R681H^ mutant (cf. Fig 4), we wished to investigate the underlying molecular mechanism of this phenomenon. Based on our results and the fact that the mutation affects a key motif of the dimer interface, we hypothesized that the mutation might perturb oligomerization of *Pf*CCT MΔK^R681H^. To reveal the potential alterations in dimer formation, mass spectrometric analysis was performed under native electrospray conditions, as an appropriate method for determining protein oligomerization state [58].

**Fig 5:**
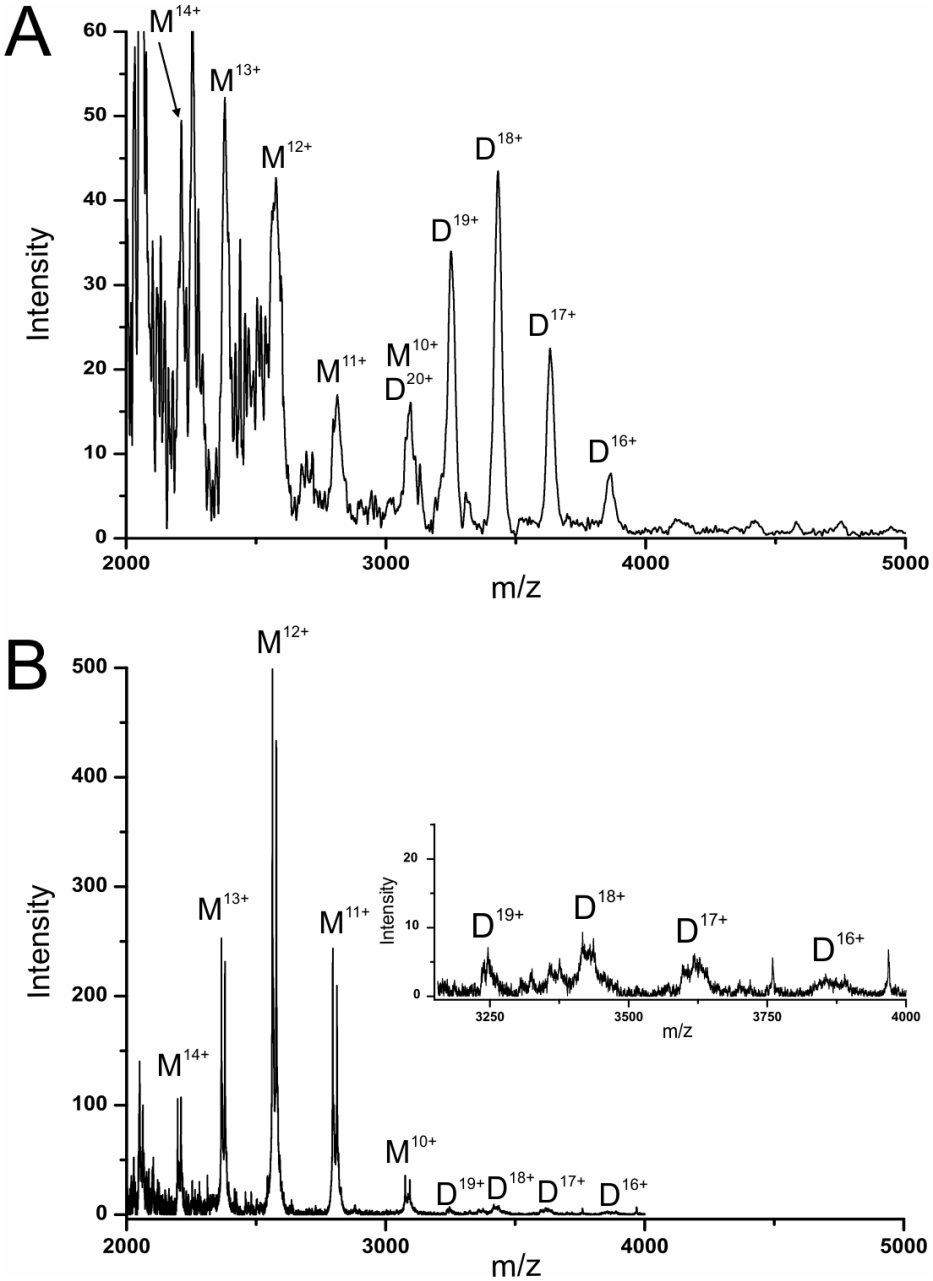
Mass spectra of *Pf*CCT MΔK^WT^ and *Pf*CCT MΔK^R681H^ proteins under native electrospray conditions. M and D indicate signals contributing monomers and dimers, respectively, while numbers denote the charge states. A) Mass spectrum of *Pf*CCT MΔK^WT^ measured in the present study for direct comparison (cf. also [10]). B) Mass spectrum of *Pf*CCT MΔK^R681H^. In the inset the 10-times enlarged graph of dimer regions (3150–4000 m/z) is shown.

Mass spectra provided well reproducible (within 20%) dimer:monomer abundance ratios from *Pf*CCT MΔK^WT^ and *Pf*CCT MΔK^R681H^. Importantly, while reasonable amount of dimer was present in the wild type enzyme (Fig 5A), the dimer:monomer abundance ratios were approximately 20 times lower in the mutant (Fig 5B), indicating attenuated dimer cohesion. These findings were also confirmed by native gel electrophoresis and glutaraldehyde crosslinking experiments (data not shown).

**Fig 6:**
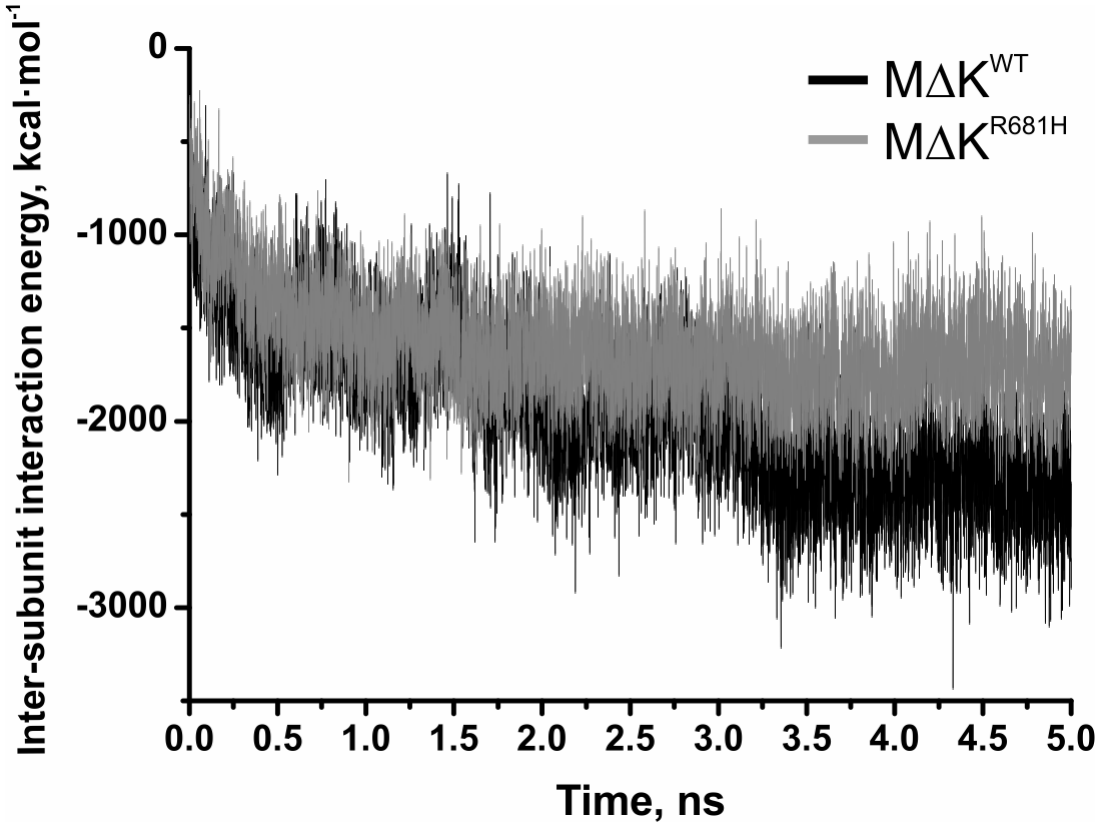
Inter-subunit interaction energies during molecular dynamics simulations. Interaction energies are calculated as Eq 1 and represented by black line in case of *Pf*CCT MΔK^WT^ and by grey line in case of *Pf*CCT MΔK^R681H^, respectively.

**Fig 7:**
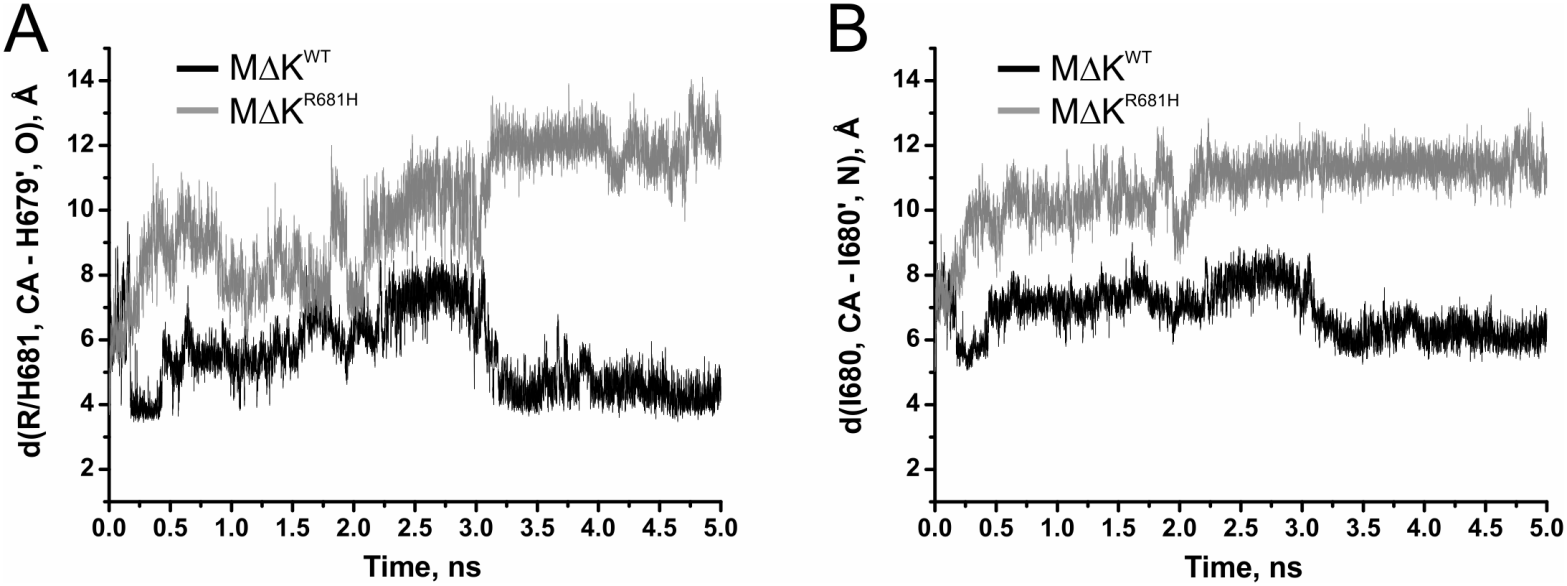
Inter-subunit interaction distances during molecular dynamics simulations. A) Variation of the atomic distance d(R/H681, CA - H679’, O) as a characteristic proximal inter-subunit contact of the catalytic domains (cf. Fig 2A and 2B). Distances are represented by black line in case of *Pf*CCT MΔK^WT^ and by grey line in case of *Pf*CCT MΔK^R681H^, respectively. B) Variation of the atomic distance d(I680, CA - I680’, N) as a characteristic proximal inter-subunit distance of the catalytic domains (cf. Fig 2A and 2B). Distances are represented by black line in case of *Pf*CCT MΔK^WT^ and by grey line in case of *Pf*CCT MΔK^R681H^.

To further analyse the effect of the mutation on binding energetics and dimer stability, molecular dynamics simulations were performed on the homology models of *Pf*CCT MΔK^WT^ and *Pf*CCT MΔK^R681H^. Productive MD simulations were carried out using an implicit solvent model at 310 K for 5 ns after a 100 ps long equilibration. Interaction energies were equilibrated at 3.25 ns resulting in a 25 percent decrease in the average inter-chain interaction energy in case of the mutant enzyme (Fig 6 and Table 3). The observed tendencies of interaction analysis reveal multiple causes possibly leading to this phenomenon. These involve much favourable solvation energy ($\Delta G_{solv}^{eq}$) of *Pf*CCT MΔK^WT^, while the van der Waals-type hydrophobic interactions ($E_{vdW}^{eq}$) also give better contributions to the effective energies of the *Pf*CCT MΔK^WT^ dimers. Thus, the interacting surface area ($A_{\text{int}}^{lf}$) of the homodimers containing mainly hydrophobic amino acid side chains is much larger in case of *Pf*CCT MΔK^WT^ leading to a more compact volume ($V^{lf}$) (Table 3). Impaired interaction of *Pf*CCT MΔK^R681H^ monomers is particularly apparent at the ^681^RWVD^684^ conserved dimer interaction motifs. To illustrate this, we followed the distance variation of two representative inter-chain distances (R/H681, CA - H679’, O and I680, CA - I680’, N) in the course of the MD simulations (cf. Fig 2). Importantly, the former interaction constitutes the proximal inter-monomer contact within the *Pf*CCT homology model as well as in the rat CCT structure [25]. The characteristic deviation of cognate distances between *Pf*CCT MΔK^R681H^ and *Pf*CCT MΔK^WT^ shown on Fig 7 argues for a major perturbation of local contacts that could contribute to observed reduction of dimer interaction surface.

**Table 3:** Overall effective interaction energy ($W_{\text{int}}^{eq}$) and the contribution of non-bonded van der Waals ($E_{vdW}^{eq}$) and Coulomb ($E_{Cou}^{eq}$) interaction energy terms and of the solvation free energy change upon dimerization ($\Delta G_{solv}^{eq}$) averaged over all frames of the equilibrated phase of the productive MD simulations.

	$W_{\text{int}}^{eq}$(kcal·mol^-1^)	$\Delta G_{solv}^{eq}$ (kcal·mol^-1^)	$E_{vdW}^{eq}$(kcal·mol^-1^)	$E_{Cou}^{eq}$ (kcal·mol^-1^)	$N_{H-b}^{eq}$	$V^{lf}$ (Å^3^)	$A^{lf}$ (Å^2^)	$A_{\text{int}}^{lf}$ (Å^2^)
***Pf*CCT MΔK** ^**WT**^	-2315 ± 266	-2777 ± 275	-188 ± 8	654 ± 54	4.0	61064	17057	1814
***Pf*CCT MΔK** ^**R681H**^	-1729 ± 233	-1973 ± 244	-141 ± 7	383 ± 58	3.0	61428	17339	1383

The superscript ‘eq’ denotes the equilibrated phase (final 1.75 ns) of the productive MD simulations. Data from the last frame of the simulations are denoted as ‘lf’. Important geometric data such as volume (($V^{lf}$) and surface area ($A^{lf}$), interaction surface area ($A_{\text{int}}^{lf}$) and the average number of inter-subunit hydrogen bonds during the simulations ($N_{H-b}^{eq}$) are also indicated.

It should be kept in mind that the entropy loss due to decrease of translational and rotational degrees of freedom opposes the formation of the dimer and this become more pronounced with increase in the temperature. This taken together with the reduced interaction energy in the mutant may explain the temperature dependence of the dimerization state of the mutant [59]. *In silico* results thus indicate the adverse effect of R681H mutation on dimer stability. Regarding the considerable extent of intersubunit interface located between dimer pair of catalytic domains, we suppose that the mutation might affect overall structural stability of the full length *Pf*CCT protein as well through perturbed interdomain interactions.

## Discussion

Our results suggest that an intact dimer form of the catalytic domains of *Pf*CCT is critical for its enzymatic function. This is in agreement with preferential dimer functional assembly of evolutionarily related cytidylyltransferases: CCT, GCT (CTP:glycerol-3-phosphate cytidylyltransferase), ECT (CTP:ethanolamine phosphate cytidylyltransferase) [60], demonstrated by multiple observations. Gene disruption experiment of *Plasmodium berghei cct* gene, a close homologue of the *pfcct* gene evolved by gene duplication revealed that the truncated protein devoid of second catalytic domain could not restore the function of the wild type form [12]. This effect is possibly mediated by the disruption of the pseudo-heterodimer interface. Analyses of crystal structures have shown that the well-studied bacterial representative of the cytidylyltransferase enzyme family *Bacillus subtilis* GCT and the mammalian representative rat CCT, which are structurally related cytidylyltransferases but only possess one CT domain each, form homodimers [25,28,61]. Cross-linking studies of the rat CCT indicated that domains N and C of rat CCT, approximately corresponding to the construct PfCCT MΔK, have a predominant contribution to dimerization. It was also shown that membrane binding, which induces enzyme activation, perturbs the dimer interface, yet it does not cause dimer dissociation [27].

The equivalent of RYVD motif, (R/K)(Y/W)VD is a general signature sequence in cytidylyltransferases [54,62] found at dimer interface of crystal structures (PDB ID: 1COZ, 3HL4 and 3ELB for *Bs*GCT, rat CCT and human ECT, respectively) [25,28]. The functional role of this conserved arginine at the dimer interface was also assessed in *Bs*GCT by alanine mutagenesis of the corresponding residue R63 resulting in a 10-fold decrease in k_cat_ values, but K_M, CTP_ was not altered considerably [62]. These results also indicate that this conserved residue does not interfere with substrate/ligand binding at the active site. Contribution of the C-terminal CT domain to the structural stability in the two tandem catalytic domain containing ECT was also suggested [63]. Identification of a novel splice variant (Pcyt2γ) lacking the C-terminal CT domain and being completely devoid of enzyme activity proved also that both cytidylyltransferase domains are required for activity [64]. The effect of a single point mutation on structural stability and protein functionality is also not without precedent in other enzyme families, as an R/H exchange in crystallins was shown to be responsible for congenital cataract through disrupted interactions at the inter-subunit interface [65].

## Conclusions

Our results reveal that R/H mutation of a conserved residue at the dimer interface does not directly compromise the enzyme activity of *Pf*CCT. Instead, it induces decreased thermal stability which in turn results in the inactivation of the enzyme. The structural model, molecular dynamics simulations as well as oligomerization results together reveal attenuation of dimer interactions induced by the point mutation. We conclude that maintaining intact dimer interactions is critical for enzyme activity of *Pf*CCT. These consequences also provide an explanation for the observed thermo-sensitive phenotype of CHO MT58 cell line, where an accelerated degradation of CCT was observed at higher temperatures.

## Supporting Information

S1 File Validation of the homology models. (PDF)

S2 File *In vivo* evaluation of protein stability. (PDF)
