# Correction: Molecular Mechanism for the Thermo-Sensitive Phenotype of CHO-MT58 Cell Line Harbouring a Mutant CTP:Phosphocholine Cytidylyltransferase

**DOI:** 10.1371/journal.pone.0165871

**Published:** 2016-10-27

**Authors:** Lívia Marton, Gergely N. Nagy, Olivér Ozohanics, Anikó Lábas, Balázs Krámos, Julianna Oláh, Károly Vékey, Beáta G. Vértessy

The following information is missing from the Funding section: This study was also funded by Research and Technology Innovation Fund (VKSZ-12-1-2013-0001).
