# Supplementary material for: Molecular Mechanism for the Thermo-Sensitive Phenotype of CHO-MT58 Cell Line Harbouring a Mutant CTP:Phosphocholine Cytidylyltransferase
Source: PLoS One. 2015 Jun 17;10(6):e0129632. doi: 10.1371/journal.pone.0129632 (PMC4470507; doi:10.1371/journal.pone.0129632)
Supplement: S1 File — (PDF) [file pone.0129632.s001.pdf]

S1 File

Validation of the homology models

Homology models of *Pf*CCT MΔK^{WT} and *Pf*CCT MΔK^{R681H} were built based on the template sequence of rat CCT (PDB ID: 4MVC). Alignments for exact sequences used for *in silico* and *in vitro* studies are shown in Fig. 1 A and Fig. S1 A, respectively. Good match of the *Pf*CCT MΔK^{WT} homology model and the template is shown when structures are overlaid (Fig. S1 B).

In order to ensure the reliability of a homology model, it is necessary to follow a strict validation protocol. Therefore, the selected homology models were evaluated using the PROCHECK program to produce the Ramachandran plots, the WHAT_CHECK algorithm was used for checking the stereochemical parameters of the residues in the models and ERRAT program for the statistical analysis of the non-bonded interactions between different atom types.

Figure S2 shows the Ramachandran plots of the template and models. In an optimal case the more than 98% of the residues should be in the favored regions with less than 0.2% outliers [1,2]. The results obtained by the PROCHECK program shown in Table S1 also confirm that none of the models nor the template suits this requirement, but the percentages are comparable between the template and models, increasing the reliability of the models. S611 of both models and S592 of the *Pf*CCT MΔK^{WT} model are in the disallowed regions. The corresponding template residue for S611 is a G, which has no side chain and therefore can adopt phi and psi angles in all four quadrants of the Ramachandran plot. The distance between the alpha C atoms of S592 and the mutated R681 is more than 23 Å in the *Pf*CCT MΔK^{WT} model and being in the only one disordered region of the protein based on the prediction of IUPred [3].

Table S2 summarizes the results of the WHAT_CHECK program. In general the achieved scores of the homology models are similar to the template structure. The Z-score of backbone

conformation is low both in the template structure and models, while improper dihedral distribution, bond lengths and angles are characterized by better scores in the model. Figure S3 demonstrates the results of ERRAT program. This algorithm plots an error function of position of a 9-residue sliding window contains statistic of non-bonded interactions between different atom types compared with highly refined structures. The "overall quality factor" shows the percentage of the protein for which the calculated error value falls below the 95% statistical rejection limit. As expected, the ERRAT scores are lower in the case of models than the template structure which is not surprising since unrefined structures generally have lower scores, and it was shown that ERRAT seems to systematically give lower scores for homology models created with MODELLER [4]. Two problematic areas are shown in the diagrams of the models: the N-terminal region which is a disordered region of the proteins as discussed below and internal part of the sequences which are responsible for the linkage of structural motifs. Despite the limited sequence identity between rat CCT and *Pf*CCT, the modelled folding of the N-terminal segment in the *Pf*CCT structure still follows the template structure remarkably well as it forges main-chain interactions to the Rossmann-folded catalytic domain core.

Supporting Figure legends

S1Fig. Sequence alignment of rat CCT and *Pf*CCT MΔK^{WT} and superimposition of the *Pf*CCT MΔK^{WT} homology model superimposed on the template (rat CCT, PDB ID: 4MVC). A) Alignment of rat CCT (PDB ID: 4MVC) and *Pf*CCT MΔK^{WT} sequences used for *in vitro* studies. Secondary structure elements are represented by squiggles (α-helices) and arrows (β-strands). In the aligned sequences, red box with white character indicates strict identity and red character means similarity in groups. The layout with secondary structure elements was generated with ESPript 3.0. B) Homology model of *Pf*CCT MΔK^{WT}

superimposed on rat CCT (PDB ID: 4MVC) template. The homology model is coloured in red and the template is coloured in blue.

S2Fig. Ramachandran plots of the template (rat CCT PDB ID: 4MVC) and of the homology models. Backbone dihedral angle pairs (Phi and Psi) are shown in the most favoured regions (red), in the additional allowed regions (yellow), in the generously allowed regions (egg-shell-white) and in the disallowed regions (white). The Ramachandran plots of the homology models are similar to the template.

S3Fig. ERRAT diagrams. The program gives a statistical analysis of the non-bonded interactions between different atom types. Lower error values indicate more favourable interactions. Rat CCT PDB ID: 4MVC *On the error axis, two lines are drawn to indicate the confidence with which it is possible to reject regions that exceed that error value. **Expressed as the percentage of the protein for which the calculated error value falls below the 95% rejection limit.**Table S1. Summary of the PROCHECK results** including the distribution of the residues in the Ramachandran plot, the number of bad contacts, and the suitability of measured and calculated (M/C) bond lengths and angles, and planarity of planar groups.

S1 Table. Summary of PROCHECK results.

S2 Table. Summary of the WHAT_CHECK results.

Supporting Tables

S1 Table. Summary of PROCHECK results.

**Structure**	**Ramachandran plot**				**Other geometrical parameters**			
	**Most favoured regions (%)**	**Additional allowed regions (%)**	**Generously allowed regions (%)**	**Disallowed regions (%)**	**Bad contacts**	**M/C bond lengths* (%)**	**M/C bond angles* (%)**	**Planar groups* (%)**
**Rat CCT**	97.2	2.8	0.0	0.0	6	100.0	99.9	100.0
***Pf*CCT MΔK^{WT}**	94.1	4.7	0.0	1.2	5	99.5	95.3	100.0
***Pf*CCT MΔK^{R681H}**	95.3	4.1	0.0	0.6	5	99.6	95.1	100.0

S2 Table. Summary of the WHAT_CHECK results.

	**Rat CCT**	***Pf*CCT MΔK^{WT}**	***Pf*CCT MΔK^{R681H}**
**Structure Z-scores** (positive is better than average)			
2nd generation packing quality	-1.909	-0.947	-1.235
Ramachandran plot appearance	-2.598	0.334	0.297
chi-1/chi-2 rotamer normality	-4.121 (bad)	0.349	0.843
Backbone conformation	-3.375 (poor)	-5.911 (bad)	-6.203
**RMS Z-scores** (should be close to 1.0)			
Bond lengths	0.232 (tight)	0.900	0.904
Bond angles	0.532 (tight)	1.209	1.210
Omega angle restraints	1.054	0.706 (tight)	0.727 (tight)
Side chain planarity	0.219 (tight)	0.308 (tight)	0.261 (tight)
Improper dihedral distribution	0.389	0.734	0.705
Inside/Outside distribution	1.086	1.086	1.095

Supporting Figures

S1 Fig.

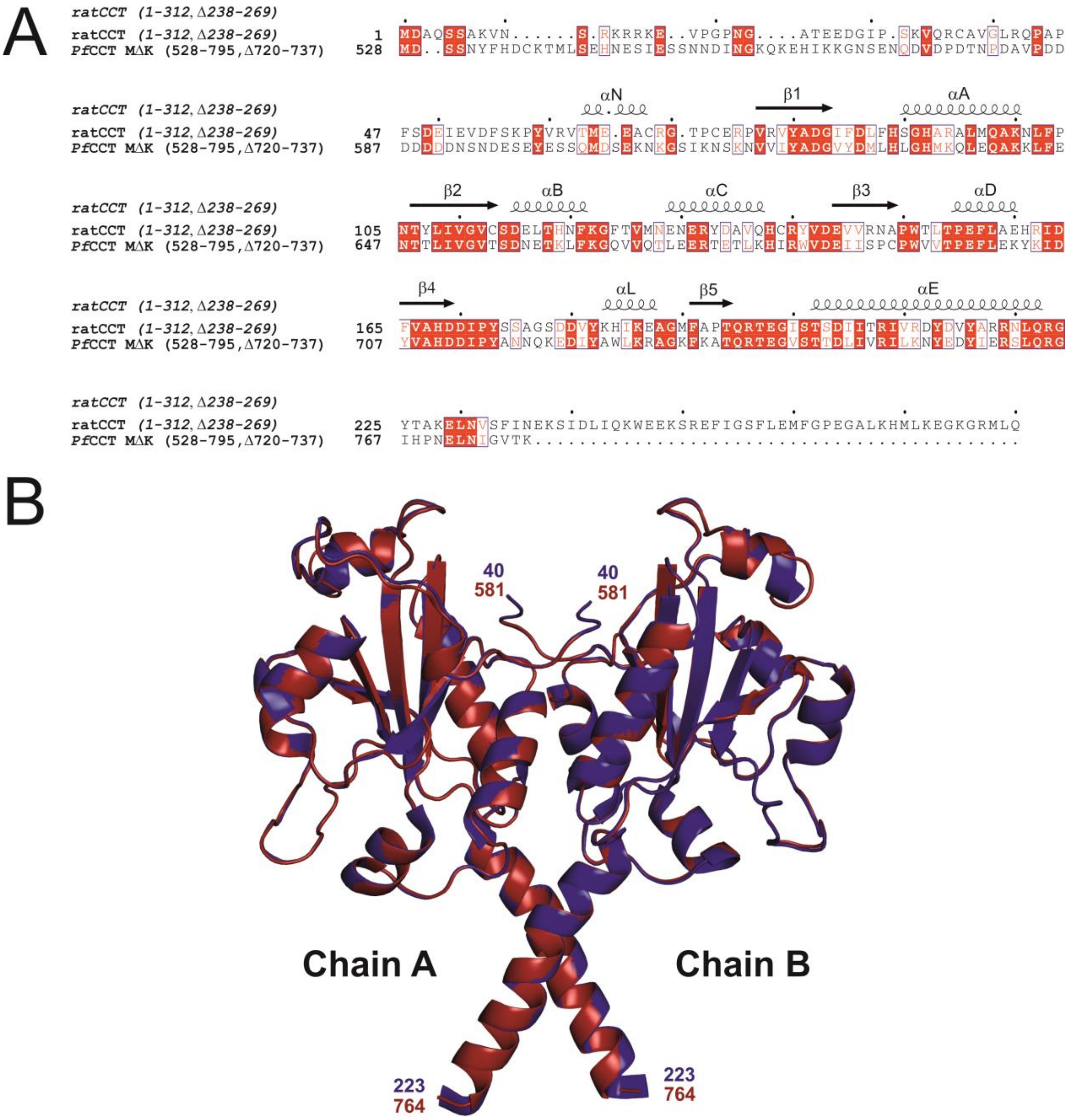

S2 Fig.

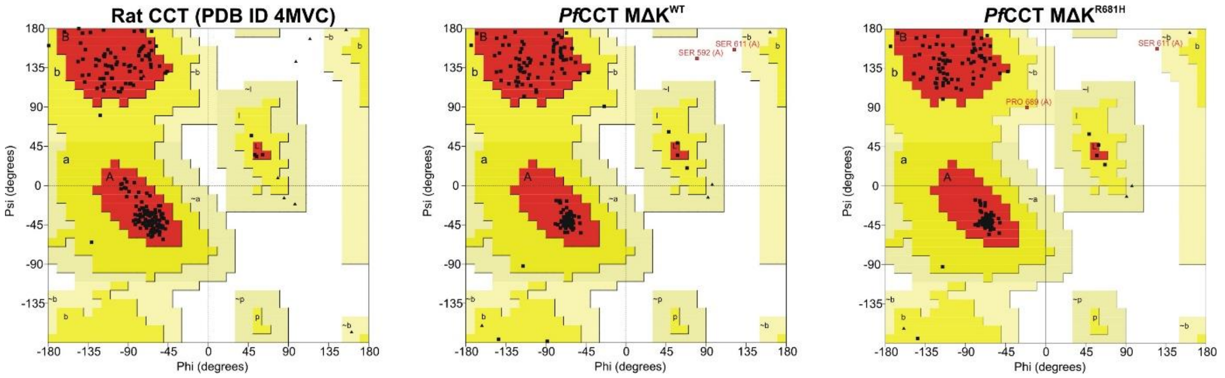
Rat CCT (PDB ID 4MVC)
PfCCT MΔK^WT
PfCCT MΔK^R681H
Psi (degrees)
Phi (degrees)
SER 611 (A)
SER 592 (A)
PRO 689 (A)

S3 Fig.

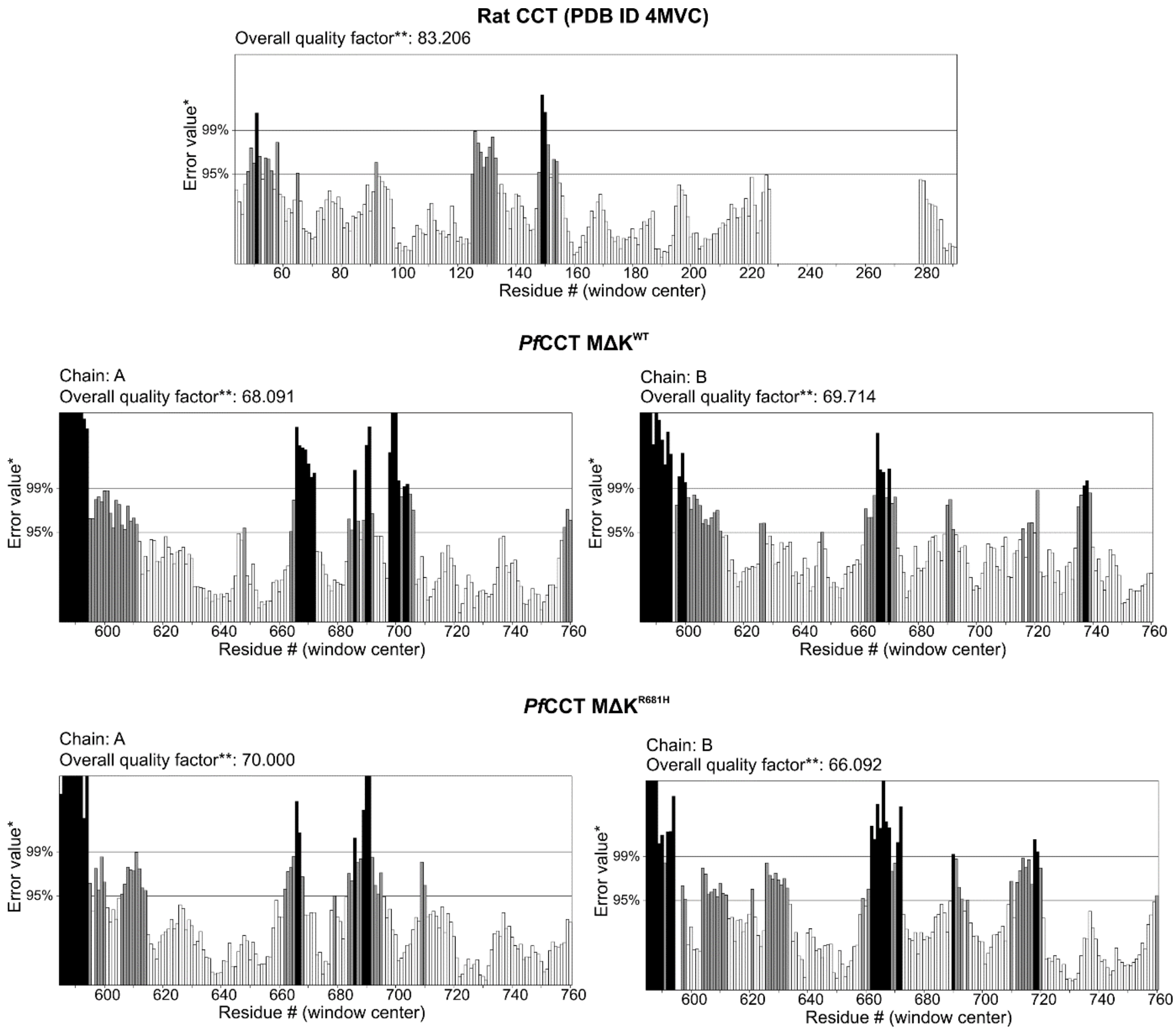
Rat CCT (PDB ID 4MVC)
Overall quality factor**: 83.206
Error value*
99%
95%
Residue # (window center)
PfCCT MΔK^WT
Chain: A
Overall quality factor**: 68.091
Chain: B
Overall quality factor**: 69.714
PfCCT MΔK^R681H
Chain: A
Overall quality factor**: 70.000
Chain: B
Overall quality factor**: 66.092

References

1. Lovell SC, Davis IW, Arendall WB, de Bakker PIW, Word JM, Prisant MG, et al. Structure validation by Calpha geometry: phi,psi and Cbeta deviation. Proteins. 2003;50: 437–50. doi:10.1002/prot.10286

2. Chen VB, Arendall WB, Headd JJ, Keedy D a, Immormino RM, Kapral GJ, et al. MolProbity: all-atom structure validation for macromolecular crystallography. Acta crystallographica Section D, Biological crystallography. 2010;66: 12–21. doi:10.1107/S0907444909042073

3. Dosztányi Z, Csizmok V, Tompa P, Simon I. IUPred: web server for the prediction of intrinsically unstructured regions of proteins based on estimated energy content. Bioinformatics (Oxford, England). 2005;21: 3433–4. doi:10.1093/bioinformatics/bti541

4. Wallner B, Elofsson A. All are not equal: a benchmark of different homology modeling programs. Protein science : a publication of the Protein Society. 2005;14: 1315–27. doi:10.1110/ps.041253405
