# Supplementary material for: Molecular Mechanism for the Thermo-Sensitive Phenotype of CHO-MT58 Cell Line Harbouring a Mutant CTP:Phosphocholine Cytidylyltransferase
Source: PLoS One. 2015 Jun 17;10(6):e0129632. doi: 10.1371/journal.pone.0129632 (PMC4470507; doi:10.1371/journal.pone.0129632)

S2 File

In vivo evaluation of protein stability

In order to investigate the *in vivo* stability of *Pf*CCT MΔK^{WT} and *Pf*CCT MΔK^{R681H} constructs, test expressions were performed in BL21 (DE3) Rosetta *E. coli* strain according to the expression protocols described in Materials and Methods. Samples before and after induction were analyzed on SDS Page. Results indicate a lower yield of *Pf*CCT MΔK^{R681H} even at 16°C (shown in Fig. S4). This experiment clearly shows lower *in vivo* stability of the mutant enzyme in *E. coli*.

Supporting Figure legends

Fig. A Test expression of the wild type and mutant enzyme in *E.coli*.

Supporting Figures

Fig. A

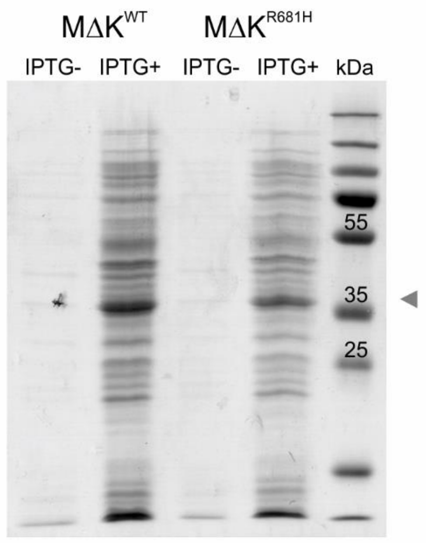

Supplement: S2 File — (PDF) [file pone.0129632.s002.pdf]
